# Diagnostic accuracy of *Pf*HRP2-based malaria rapid diagnostic tests and antigenemia persistence in Kenyan children from a holoendemic region: implications for case management and surveillance

**DOI:** 10.3389/ebm.2025.10585

**Published:** 2025-05-22

**Authors:** Sharley A. Wasena, Clinton O. Onyango, Shamim W. Osata, Samuel B. Anyona, Evans Raballah, Ivy Hurwitz, Philip D. Seidenberg, Collins Ouma, Qiuying Cheng, Kristan A. Schneider, Douglas J. Perkins

**Affiliations:** ^1^ University of New Mexico-Kenya Global Health Programs, Kisumu, Kenya; ^2^ Department of Biomedical Sciences and Technology, School of Public Health and Community Development, Maseno University, Maseno, Kenya; ^3^ Department of Medical Biochemistry, School of Medicine, Maseno University, Maseno, Kenya; ^4^ Department of Medical Laboratory Sciences, School of Public Health, Biomedical Sciences and Technology, Masinde Muliro University of Science and Technology, Kakamega, Kenya; ^5^ Center for Global Health, Department of Internal Medicine, University of New Mexico, Albuquerque, NM, United States; ^6^ Department of Emergency Medicine, School of Medicine, University of New Mexico, Albuquerque, NM, United States

**Keywords:** malaria diagnosis, *Plasmodium falciparum*, histidine rich protein, *Pf*HRP2, mRDT

## Abstract

Malaria remains a significant cause of childhood morbidity and mortality, with *Plasmodium falciparum* Histidine-Rich Protein 2 (*Pf*HRP2)-based malaria rapid diagnostic tests (mRDTs) widely used in endemic regions where microscopy is sometimes not feasible. While these tests offer high sensitivity, persistent *Pf*HRP2 antigenemia and gene deletions can cause false-positive and false-negative results, compromising their accuracy for malaria case management and surveillance. This study evaluated the diagnostic performance and antigen persistence of *Pf*HRP2-mRDTs using data from a longitudinal birth cohort of 750 children followed monthly from birth to 36 months in a holoendemic region of Kenya. Malaria diagnosis was performed using both microscopy and mRDTs, with a total of 15,006 clinical events recorded from 573 children between 2017 and 2023. Data from an independent acute febrile cohort of 937 children (<5 years) followed for 14 days was analyzed to validate the findings. The mRDT showed a high sensitivity of 97.27% but a moderate specificity of 65.00% in acute febrile illness, indicating frequent false-positive results. The positive predictive value was low (35.10%), suggesting that confirmatory testing is needed, while the negative predictive value was high (98.89%), reinforcing the reliability of mRDTs in ruling out malaria. Persistent *Pf*HRP2 antigenemia was observed, with a median antigen clearance time of 51.14 days, respectively. Higher initial parasite densities (>50,000/μL) were associated with a slower antigen decay rate (*p* = 0.001), highlighting the challenge of interpreting positive mRDT results after treatment. Validation using the acute febrile cohort showed that mRDT specificity exceeded 95% at initial diagnosis and follow-up. Overall, *Pf*HRP2-based mRDTs remain valuable for frontline malaria diagnosis but are limited by antigen persistence, leading to false positives in follow-up testing. Where feasible, integration of confirmatory diagnostic methods, such as microscopy or molecular assays, could improve the performance of malaria case management and clinical decision making, particularly in high-transmission settings.

## Impact statement

The findings from this study provide critical insights into the diagnostic performance and antigen persistence of PfHRP2-based malaria rapid diagnostic tests (mRDTs) in a holoendemic region, highlighting their strengths and limitations in clinical and surveillance settings. The study advances the field by demonstrating that while mRDTs have high sensitivity and reliability in ruling out malaria, their specificity is compromised due to prolonged antigenemia, leading to false positives during follow-up assessments. This work presents novel longitudinal data showing that antigen persistence extends beyond two months post-treatment, with higher initial parasite densities slowing clearance. These findings have significant implications for malaria case management, emphasizing the need for confirmatory diagnostic strategies to improve accuracy and treatment decisions. By identifying antigenemia persistence as a key challenge, this study informs malaria control programs and underscores the necessity for improved diagnostic algorithms to enhance surveillance, reduce overtreatment, and guide policy development in high-transmission regions.

## Introduction

Malaria continues to pose a major global public health threat, with 263 million cases of illness and 597,000 deaths annually [1]. In the World Health Organization (WHO) African Region, *Plasmodium falciparum*, the deadliest malaria species, accounts for the majority of the cases (247 million, 94%) and mortality (567,000, 95%) [1]. Children under five are the most vulnerable group, accounting for 76% of the malaria-related fatalities (432,400) in this region [1–3]. Kenya faces a substantial challenge, with ∼3.29 million cases and 1,060 deaths annually, primarily in this susceptible age group [1]. In western Kenya, the highest-burden area, malaria prevalence is exceptionally high in Siaya County, where the microscopy positivity rate among children under five was reported to be 54.9% [4]. Another survey in western Kenya found a positivity rate of 32.8% among children under five, with prevalence increasing with age [5]. These findings highlight the disproportionate impact of malaria on young children in western Kenya, emphasizing the need for targeted interventions and prevention measures to reduce morbidity and mortality in this high-risk population.

Achieving these goals requires accurate diagnosis and timely treatment, which are critical for effective malaria management [6]. Misdiagnosis or delays in treatment can result in severe complications and increased mortality rates [3, 6]. To address this, the WHO recommends the use of parasite-based diagnostic methods, such as microscopy and/or malaria rapid diagnostic tests (mRDTs) before administering antimalarial treatment [7]. Moreover, residual drug concentrations in misdiagnosed individuals can shape a selective environment facilitating the evolution of drug resistance [8, 9].

Microscopy is the gold standard for malaria diagnosis, providing highly sensitive and specific detection of malaria parasites, allowing for quantifying parasitemia and identifying the infecting *Plasmodium* species, which is crucial for determining appropriate treatment strategies [3, 7, 10]. Despite these advantages, microscopy faces significant challenges in resource-limited settings. The method requires personnel with expertise in preparing and interpreting blood smears, along with access to specialized equipment, high-quality reagents, and a reliable electricity supply. These infrastructure and resource constraints limit microscopy’s availability and consistent application in many endemic regions [10, 11].

Consequently, alternative diagnostic tools, such as mRDTs have become essential for improving access to cost-effective malaria diagnosis in resource-limited settings [12, 13]. The mRDTs are immuno-chromatographic tests that detect parasite-specific antigens in peripheral blood, typically using a finger-prick blood sample [7]. Compared to microscopy, mRDTs are relatively simple to perform and interpret, overcoming the challenges of infrastructure limitations and making them valuable in resource-constrained areas [7, 14]. The mRDTs vary in their ability to detect different malaria species (e.g., *P. falciparum*, *P. vivax*, *P. ovale*, and *P. malariae*) by targeting specific antigens produced by the malaria parasites, enabling the differentiation of species or confirming the presence of *Plasmodium* parasites in mixed infections [7, 10]. For *P. falciparum*, the commonly targeted antigen is histidine-rich protein 2 (*Pf*HRP2) with some cross-reactivity with *Pf*HRP3 [10, 15]. Other mRDTs detect the enzyme *Plasmodium* lactate dehydrogenase (*P*LDH), which is expressed by all *Plasmodium* species but can be species-specific depending on the test [10]. Additionally, aldolase, another enzyme involved in the parasite’s glycolytic pathway, may be targeted for broader detection of non-*P. falciparum* species [10].

However, challenges such as *Pf*HRP2 gene deletions can impact the reliability of mRDTs in diagnosing *P. falciparum* infections and provide false negative results [3, 15]. Such deletions have been reported in multiple malaria-endemic areas, including Kenya, although with a low prevalence [16–18]. Infection caused by non-falciparum species can also result in false negatives since *Pf*HRP2-based mRDT is designed to detect *P. falciparum*-specific antigens [19]. In addition, low-density parasitemia below the detection threshold (100-200 parasites/µL) [20] of mRDTs can decrease the sensitivity of *Pf*HRP2-specific tests and generate false negative results [21, 22].

The widespread use of *Pf*HRP2-based mRDTs for detecting *P. falciparum* in holoendemic regions (e.g., western Kenya) presents both advantages and disadvantages. While these tests offer a viable tool for prompt diagnosis in resource-constrained settings, the thermostable *Pf*HRP2 antigens can persist in the bloodstream after parasite clearance, leading to false-positive results in individuals who have cleared the parasite [23–25]. This persistent antigenemia complicates the detection of new infections after antimalarial treatment, reducing the effectiveness of mRDTs for post-treatment follow-up, particularly in high-transmission areas where recurrent malaria infections are common. Collectively, both false-negative and false-positive results can impact treatment decisions and potentially contribute to the misinterpretation and mismanagement of malaria cases.

Treating a child with antimalarials when the individual is aparasitemic can contribute to the selection and spread of drug-resistant malaria strains by exerting unnecessary drug pressure, potentially eliminating susceptible parasites while allowing resistant ones to survive and propagate [26]. In addition, treating a child for malaria when they do not have the infection can result in missing the actual cause of their symptoms, such as bacterial or viral infections that are common co-morbidities in malaria-endemic settings and require different treatments [27–29]. This can result in potentially delaying appropriate care and lead to worsening of the child’s condition or complications from the untreated underlying illness. Antimalarial drugs can also have side effects, including gastrointestinal symptoms and allergic reactions and, in rare cases, severe adverse effects like neurotoxicity or cardiac issues [30, 31]. Unnecessary administration of these drugs exposes the child to these risks without any therapeutic benefit.

In the current study, we utilized concurrent microscopy and mRDTs for diagnosis of malaria infection in a mother-child birth cohort followed for 36 months (mos.), aimed at improving malaria management in affected cases. The longitudinal design provided valuable insights into the sensitivity and specificity of these diagnostic tools in detecting malaria in a highly vulnerable population of young children. Repeated longitudinal measurements allowed for the evaluation of diagnostic methods during early childhood when children are most vulnerable to malaria, as their immunity develops through repeated infections with varying parasite levels. Additionally, the study enabled an assessment of the persistence of antigenemia detected by the *Pf*HRP2 based mRDT across multiple infection events (n = 9,958). Collectively, these findings present a comprehensive evaluation of mRDT performance in children during their first 3 years of life in a high *P*. *falciparum* transmission setting.

## Materials and methods

### Mother-child pair longitudinal birth cohort

To evaluate the performance of mRDTs (i.e., CareStart Malaria HRP2, *Pf*) in a holoendemic *P. falciparum* setting, we first utilized data collected from a prospective birth cohort that enrolled 750 mother/child pairs between July 2017 and February 2023 at the Siaya County Referral Hospital (SCRH). Details of the study area have previously been described [32]. The mother/child cohort study was designed to investigate the pathogenesis of severe malaria anemia [SMA (hemoglobin, Hb) <6.0 g/dL] during the development of naturally acquired malarial immunity over the first 36 mos. of life in HIV-negative children. Data utilized for the current investigation included measures collected from the mothers at delivery and from the children across the follow-up period. Pregnant women received an explanation of the research protocol and were provided with HIV counseling, with the understanding that enrollment decisions would be made following HIV screening using rapid serological tests. HIV-negative women, regardless of malaria status, who met the following criteria were enrolled: ≥16 years of age, capable and willing to sign informed consent, willing to enroll their child in the study, committed to attending scheduled appointments and study visits over 36 mos., able to provide two contacts familiar with the child’s whereabouts, and living within a 25-km radius from the hospital. The study was approved by the Institutional Review Board of the University of New Mexico, United States, and the Maseno University Scientific and Ethics Review Committee, Kenya.

### Follow-up of children in the longitudinal birth cohort

**FIGURE 1:**
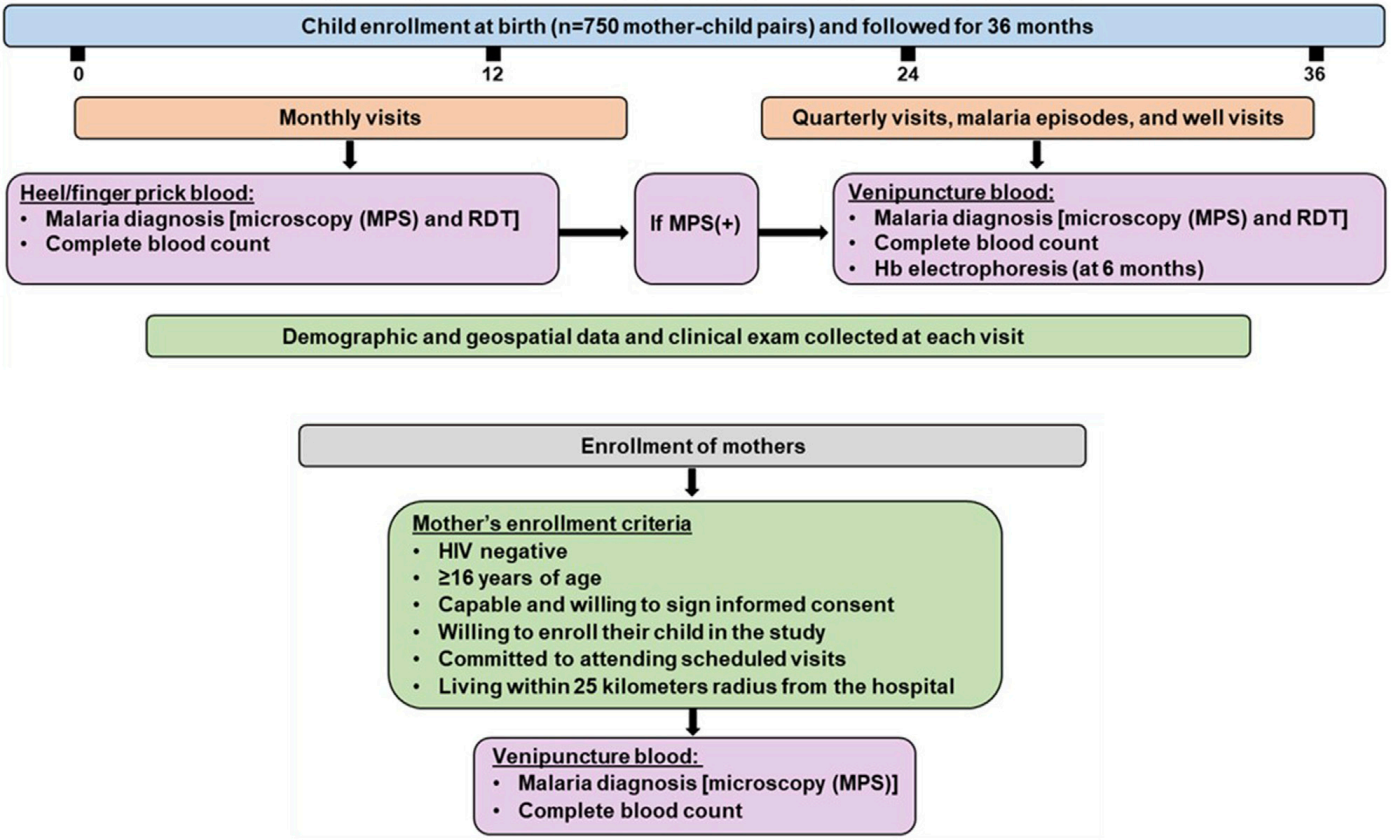
Mother-child longitudinal cohort study design. The study was conducted at Siaya County Referral Hospital, a holoendemic *P. falciparum* transmission region. Infants were enrolled at birth (n = 750) and followed for 36 months. Malaria and other endemic infections were monitored monthly and at acute febrile episodes throughout the study. Monthly testing for malaria was performed using microscopy and mRDT in heel/finger prick blood samples. Quarterly testing for malaria was conducted using microscopy and mRDT in venipuncture blood. Venipuncture was also performed on children found to have malaria at monthly or acute febrile visits and a 14-day follow-up (well-visit). Demographic and geospatial data were collected at each visit and a complete physical exam was performed. The study included HIV-negative mothers who met the inclusion criteria, regardless of malaria status. MPS: Malaria parasite, mRDT: malaria rapid diagnostic test, (+): positive, (−): negative.

Children were followed monthly for 36 months. Comprehensive demographic data was collected, and a complete physical exam was performed at each scheduled and non-scheduled (acute) visit. If parents/guardians missed a scheduled visit, study staff visited the residence to check the child’s health status (including mortality). Children were readily located through our geospatial surveillance system, which captured the participants’ location at enrollment when mothers/guardians were provided transport to their residences/homesteads. To monitor infections and hematological parameters, heel/finger-prick blood (<100 μL) was collected monthly, while venipuncture blood (2-3 mL) was obtained quarterly, during malaria episodes, and 14 days after a documented illness (well-check). To monitor health status, parents/guardians were provided with a thermometer (Vive Precision^®^) and asked to bring their child to the hospital when febrile (>37.5°C). To ensure comprehensive documentation of all malaria episodes and other pediatric infectious diseases during the study, all participants underwent comprehensive laboratory testing for proper diagnosis, followed by clinical management according to the Kenya Ministry of Health guidelines. The study design and information captured at the visits are presented in Figure 1.

### Acute febrile validation cohort

A prospective cohort study of children with acute febrile illness was also conducted at SCRH, enrolling 937 children aged 1–59 months between March 2017 and January 2024. Children presenting at SCRH with symptoms of infectious diseases were eligible if they met the following criteria: temporal temperature of ≥37.5°C, in-patient admission residence within 25 km of the hospital, and the parent/guardian willing to sign an informed consent and commit to a day-14 follow-up. Children with non-infectious diseases or a history of prior hospitalization were excluded from the study. Demographic and clinical data were collected at enrollment, and each child underwent a comprehensive physical examination. Peripheral blood (3–4 mL) was drawn via venipuncture before starting antimalarial treatment for hematological parameters, as well as malaria diagnosis using microscopy and an mRDT (CareStart Malaria HRP2, *Pf*).

### Laboratory procedures for longitudinal birth and acute febrile validation cohort

Complete blood counts (CBCs) were performed using a DxH 500 hematology analyzer (Beckman-Coulter; Miami, FL, United States). The mRDT (CareStart Malaria HRP2, *Pf*) was used to screen for malaria infection following the manufacturer’s protocol [33]. Briefly, 5 µL of whole blood was placed on a sample well, and two drops of buffer were added to the buffer well. The results were determined after 15–20 min at room temperature. Malaria parasite densities were determined using light microscopy as previously described [32]. Briefly, Giemsa-stained thick and thin blood smears were prepared and examined for asexual malaria parasites in 200 high-power fields under oil immersion at ×1,000 magnification (i.e., a ×100 objective lens with a ×10 eyepiece). Parasite density was determined per 300 leukocytes and estimated using the total leukocyte count for each patient as determined by the hematology analyzer. For quality control, a second well-trained laboratory personnel read and independently confirmed the malaria microscopy results. Thick blood smears were considered negative if no parasites were observed in 200 high-power fields. The thresholds of low (≤999 parasites/µL), medium (1,000–49,999 parasites/µL), and high parasitemia (≥50,000 parasites/µL) were defined by a combination of nominal and prior field studies in holoendemic transmission settings. For example, mRDTs can perform less favorably at <1,000 parasites/µL (∼0.02% parasitemia, assuming a standard RBC count of 5 M cells), whereas more severe malaria cases in our studies and others are commonly associated with parasite densities above 50,000 parasites/µL (∼1.0% parasitemia) [24, 34–38]. Sickle-cell trait status was determined by alkaline cellulose acetate electrophoresis (Helena Laboratories, Beaumont, TX, United States). In the acute febrile cohort, HIV-1 status was determined by two rapid serological antibody tests (Unigold™ Trinity Biotech, Jamestown, NY, United States and Determine™). For cases with single or concordant positive results, an HIV-1 proviral DNA PCR test was used to confirm infection [39]. Bacteremia was diagnosed by performing bacterial cultures on ∼1.0 mL of venipuncture blood collected aseptically, inoculated into BD BACTEC™ PEDS Plus PRIME Medium Culture Vials (Becton-Dickinson, Franklin Lakes, NJ, United States), and incubated in an automated BACTEC 9050 system (Becton-Dickinson) for 5 days. Positive alerts were then examined by Gram staining and sub-cultured on blood agar, chocolate agar or MacConkey agar plate (Hardy Diagnostics, Santa Maria, CA, United States) [27].

### Data selection for evaluating diagnostic accuracy and antigenemia persistence of the *PfHRP2*-mRDT in the longitudinal cohort

**FIGURE 2:**
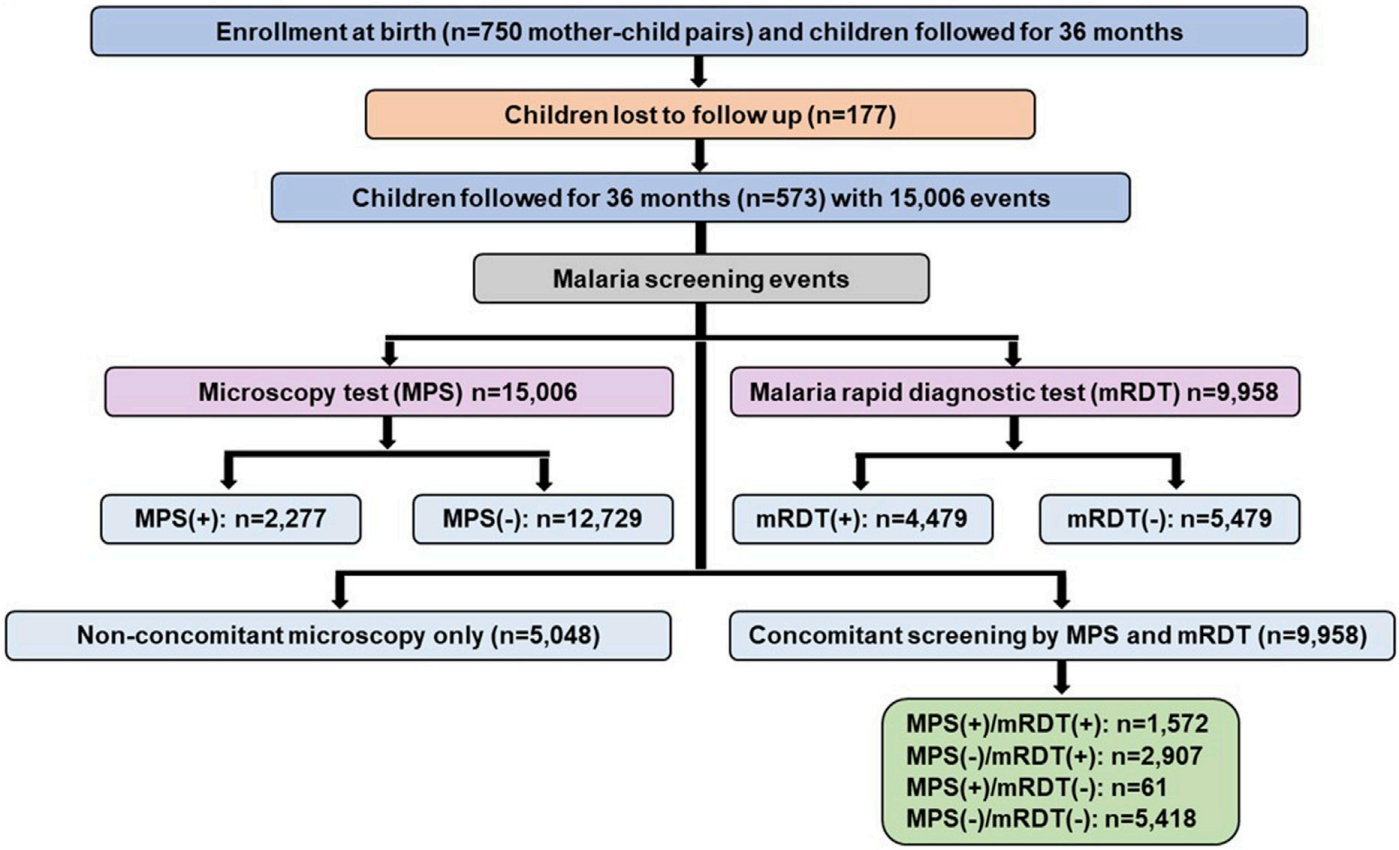
Data selection strategy for mother-child longitudinal cohort. The study enrolled 750 mother-child pairs in the longitudinal birth cohort. Based on relocation of study participants during the COVID-19 pandemic, 573 children had comprehensive follow-up information over 36 months, contributing to 15,006 clinical events. Malaria screening was performed using microscopy in all events, with 2,277 positive cases and 12,729 negatives. Malaria rapid diagnostic tests (mRDTs) were conducted for 9,958 events, with 4,479 positive and 5,479 negative results. A subset of 9,958 events had concomitant microscopy and mRDT results, classified as MPS (+)/mRDT (+) (n = 1,572), MPS (−)/mRDT (+) (n = 2,907), MPS (+)/mRDT (−) (n = 61), and MPS (−)/mRDT (−) (n = 5,418). An additional 5,048 events underwent microscopy-only screening since mRDT was introduced later in the study. MPS: Malaria parasite, mRDT: malaria rapid diagnostic test, (+): positive, (−): negative.

The mother-child cohort study enrolled 750 mother-child pairs with children followed longitudinally for 36 mos. after delivery (Figure 2). Due to the COVID-19 pandemic, there was a substantial relocation of study participants and subsequent loss to follow-up of 177 children. As such, 573 children remained in the cohort with comprehensive data for the scheduled longitudinal follow-up and acute visits, totaling 15,006 events. Of these, 2,277 were microscopy positive for malaria (MPS+) and 12,729 were microscopy negative (MPS-). From the 15,006 events for which microscopy was performed, there were 9,958 events with concomitant mRDT results. The mRDT (+) events were 4,479, while the mRDT (−) events were 5,479. All analyses presented are based on the 9,958 events with concomitant microscopy and mRDT results, enabling a direct comparison of mRDT performance against microscopy as the gold standard.

### Data selection to validate the mRDT specificities and sensitivities in an acute febrile cohort

**FIGURE 3:**
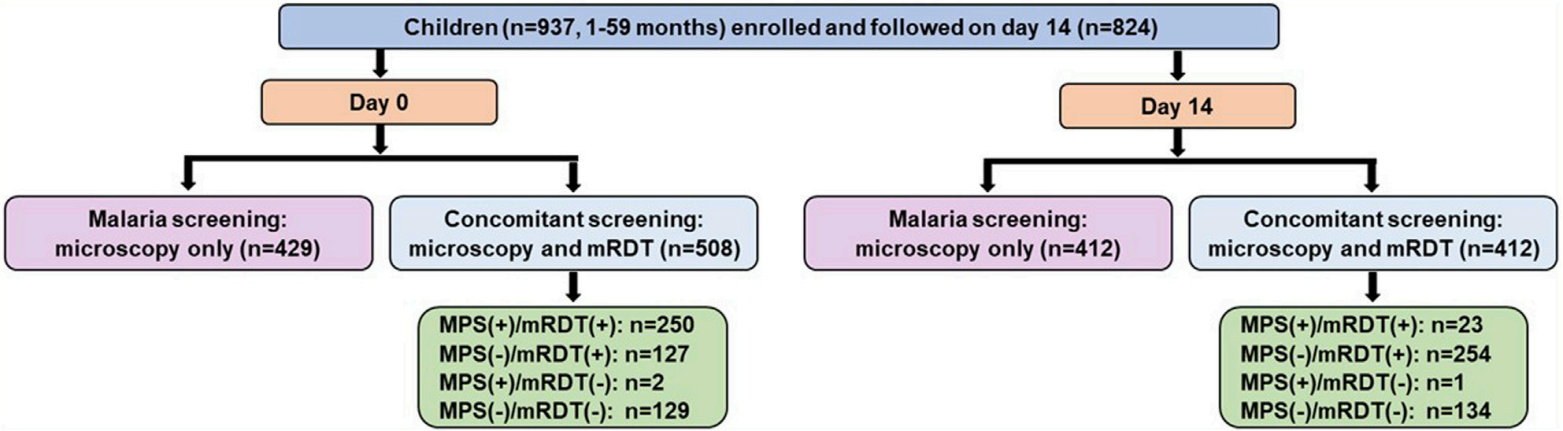
Data selection strategy for the short-term febrile cohort. Malaria screening outcomes among 937 febrile children (aged 1–59 months) enrolled in an acute febrile illness cohort and followed up on Day 14 (well-visit). At Day 0 (acute presentation, pre-treatment), malaria screening was performed using microscopy alone (n = 429) or concomitant microscopy and malaria rapid diagnostic tests (mRDT) (n = 508). Among those with concomitant screening, 250 children were positive by both microscopy and mRDT [MPS (+)/mRDT (+)], 127 were negative by microscopy but positive by mRDT [MPS (−)/mRDT (+)], 2 were positive by microscopy but negative by mRDT [MPS (+)/mRDT (−)], and 129 were negative by both tests [MPS (−)/mRDT (−)]. At the Day 14 follow-up visit, 824 children returned, with 412 undergoing microscopy-only screening and 412 receiving concomitant microscopy and mRDT testing. Among the concomitant testing group, 23 children were positive by both microscopy and mRDT, 254 were negative by microscopy but positive by mRDT, 1 was positive by microscopy but negative by mRDT, and 134 were negative by both tests. MPS: Malaria parasite, mRDT: malaria rapid diagnostic test, (+): Positive, (−): Negative.

The short-term cohort enrolled 937 children on day 0 (acute episode) and had 824 individuals return for the follow-up (well-visit) on day 14 (Figure 3). Concomitant microscopy and mRDT results were available for 508 children on day 0 and 412 on day 14.

### Statistical analysis

**FIGURE 4:**
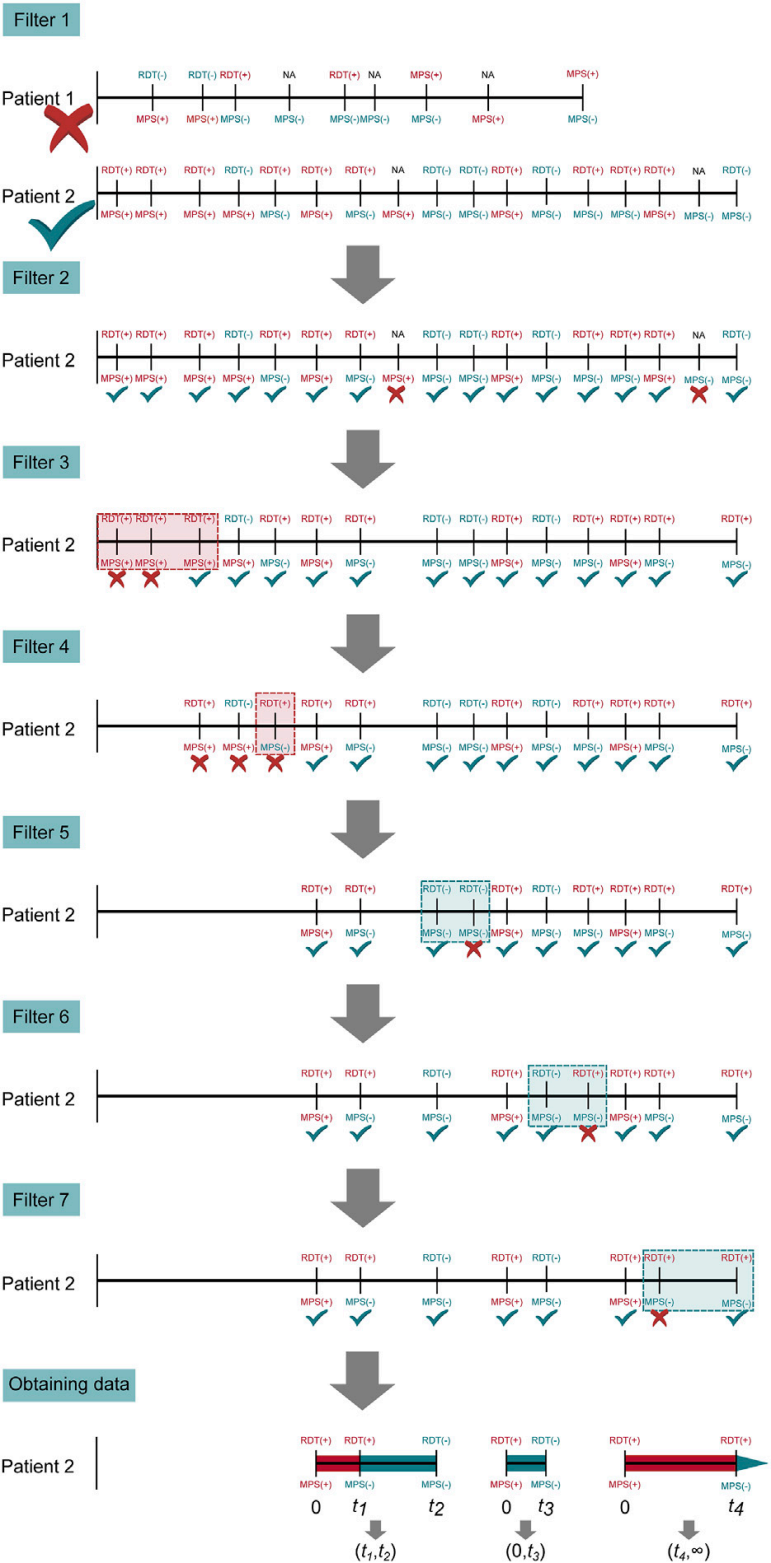
Steps of pre-processing to obtain interval-censored data. Illustrated are the seven filtering steps applied to patient visits to obtain interval-censored time-to-event data to ensure accurate evaluation of PfHRP2 antigen persistence. Filter 1: Excludes patients without both mRDT and microscopy results; Filter 2: Retains only patients with at least one positive malaria microscopy (MPS+) and mRDT (+) event; Filter 3: Removes redundant consecutive positive MPS(+) events, selecting only the last occurrence in each sequence; Filter 4: Eliminates inconsistent mRDT (−) results within an MPS(+) sequence to ensure HRP2 decay tracking; Filter 5: Retains only the first microscopy-negative (MPS-) visit following the last positive malaria episode; Filter 6: Removes intermittent mRDT-positive results in sequences where microscopy was negative; Filter 7: Final refinement ensures consistent representation of diagnostic results. The final data set captures distinct malaria infection episodes and HRP2 antigen persistence to evaluate mRDT accuracy and antigen clearance over time. Horizontal lines correspond to longitudinal follow-up periods past the enrollment date (i.e., birth). Vertical lines indicate patient visits, with MPS and mRDT results. For example, patient 1 was eliminated by the first filter since the patient had no MPS (+) and mRDT (+) result. Patient 2 has three interval-censored events, which are treated as independent observations in the parametric Cox proportional hazard model.

The demographic and clinical characteristics for concomitant microscopy and mRDT measures were analyzed using SPSS (version 23.0, SPSS Inc., Chicago, IL, United States) and R (version 4.3.0). To compare the distributions of categorical variables to null distributions, χ^2^ goodness of fit test was used. Continuous variables across groups were compared using Kruskal-Wallis test. In contrast, the Mann-Whitney U test was utilized for pairwise comparisons, particularly when the parasitemia variable had a median of zero. The sensitivity, specificity, and predictive values of the mRDT were calculated using the two-by-two contingency tables [40]. The Wilson score method was used to calculate the 95% confidence intervals for the sensitivity, specificity, Positive Predictive Value (PPV), and Negative Predictive Value (NPV) [41]. A parametric, interval-censored Cox proportional hazards model with a gamma distribution was used to evaluate *Pf*HRP2 antigenemia persistence. This distribution offers flexibility and accounts for variability in antigen clearance, allowing for non-constant hazard rates by modeling antigen decay as a multi-step process with a non-constant hazard function capturing individual heterogeneity and providing a biologically relevant estimation of antigen persistence. To obtain interval censored data, all observations need to be pre-processed by applying seven filters to the dataset to accurately analyze definitive malaria events (MPS+) in HRP2 decay across time (Figure 4). (Filter 1) Inclusion Criteria: Inclusion of subjects who were (MPS+) and mRDT (+) at least once. From these data, all visits before the first (MPS+) and mRDT (+) visit were disregarded. This approach focused the analysis on the period following the initial confirmed malaria episode. (Filter 2) Data Refinement: Only cases where an mRDT result was available were selected to ensure that the analysis was based on consistent diagnostic criteria across all participants (microscopy results were available for all visits). (Filter 3) Sequential Data Selection: To analyze HRP decay, only the last visit in sequences of consecutive MPS (+) visits was used. This strategy was used to avoid over-representation of repeated positive results, which could bias the decay analysis. (Filter 4) Consistency in Diagnostic Results: If a visit was MPS (+) but mRDT (−), the mRDT result was considered false negative. Since such outcomes cannot be used for the decay of malaria antigens, all visits from the preceding MPS (+) and RDT (+) visit to the next were excluded. This ensured that only sequences of visits with consistent positive results across both diagnostic methods were considered. (Filter 5) Handling Negative Results: For subjects who were negative by both microscopy and mRDT in consecutive visits, only the first of these visits was retained. This eliminated redundant negative visits, focusing on the first occurrence of a negative result in each sequence. (Filter 6) Addressing Inconsistent mRDT Results: In cases where a subject was intermittently mRDT (−) and MPS (−) between two mRDT (+) but MPS (−) visits, only the visits up to (including) the first mRDT (−) result were considered. Subsequent visits in such sequences were excluded to maintain the focus on initial malaria clearance. (Filter 7) Selecting the Relevant Endpoint in a Sequence: If a sequence began with an mRDT (+) and MPS (+) visit, and was followed by consecutive mRDT (+) and MPS (−) visits, only the final mRDT (+) and MPS (−) visit in the sequence was retained. This approach focused on the period directly associated with *Pf*HRP decay and malaria clearance. After applying these filters, it was possible to define intervals in which the parasite antigen decayed. As such, an mRDT (+) and MPS (+) visit at time t_0_ is either followed by (i) an mRDT (−) and MPS (−) event at time t_1_, in which case the antigen decayed in the interval (t_0_, t_1_); first an mRDT (+) and MPS (−) and second an mRDT (−) and MPS (−) visit at times t_1_ and t_2_, in which case the antigen decayed in the interval (t_1_, t_2_); or (iii) and first an mRDT (+) and MPS (−) and second an mRDT (+) and MPS (+) visit at times t_1_ and t_2_, in which case the antigen decayed in the interval (t_1_, ∞). Antigen decays between malaria episodes of the same patient were considered independent, as the analysis focused on the decay rather than the patient. The model was fitted separately: (i) without covariates, (ii) with stratification based on initial parasite densities, and (iii) sickle cell genotypes. All *p*-values ≤0.05 were deemed statistically significant.

## Results

### Maternal and child characteristics in the longitudinal cohort

The demographic characteristics of individuals in the mother-child birth cohort (n = 573 mothers and n = 573 children) are presented in Table 1. The median age for the mothers was 23.3 years, with gravidity differing among the women (*p* = 3.067E-51). The majority of the cohort had normal vaginal (92.3%) and singleton (97.9%) deliveries with 19.9% of the women experiencing preterm labor (<37 weeks). There was a higher proportion of male (55.5%) offspring than female (44.5%) in the cohort (*p* = 0.010). HbS genotypes were found to be in Hardy-Weinberg equilibrium in the study population (*p* = 0.492), but the prevalence of sickle cell trait [HbAS (16.2%)] and sickle cell disease [HbSS (1.1%)] was higher and significantly different from populations in other high-transmission regions in central and western Africa [42–45]. Throughout the 36 mos. follow-up, there were 15,006 child contact visits, the majority of which were afebrile (91.3%, n = 13,704), with 8.7% (n = 1,302) being febrile (*p* ≤ 2.200E-16). The median Hb concentration across the follow-up period was 10.58 g/dL. During the contact visits, 15.2% (n = 2,277) were malaria-positive based on light microscopy on thick and thin blood smears. The overall distribution of low (17.5%, n = 399), medium (65.7%, n = 1,496), and high (16.8%, n = 382) density parasitemia was not uniform (*p* = 1.972E-260), with the majority of the 2,277 microscopy-positive events being of medium density. The geometric mean parasite density for positive blood smears was 7,303.6 MPS/µL. Nearly all microscopy-confirmed cases had single-species infections with *P. falciparum* (99.3%, n = 2,261), followed by *P. malariae* (0.6%, n = 13). Mixed-species infections with *P. falciparum* and *P. malariae* (0.1%, n = 3) were rare, and the distribution of infections was not uniform (*p* ≤ 2.200E-16). *P. falciparum* specific HRP2 based malaria rapid diagnostic test (mRDT) results were performed for 66.4% (n = 9,958) of the total events.

**TABLE 1:** Child and maternal characteristics in the mother-child cohort study.

Characteristic		*p*-value
Maternal Factors		
Number of mothers, n (%)	573 (100)	
Age (years)	23.3 (7.8)	N/A
Gravidity, n (%)		
Primigravidae	232 (40.5)	**3.067E-51** ^a^
Secundigravidae	126 (22.0)	
Multigravida (≥3)	215 (37.5)	
Type of delivery, n (%)		
Normal birth	529 (92.3)	**≤2.200E-16** ^a^
Cesarian birth	44 (7.7)	
Twins, n (%)	12 (2.1)	N/A
Preterm labor (<37 weeks), n (%)	114 (19.9)	N/A
Childhood Factors		
Number of children, n (%)	573 (100)	
Sex, n (%)		
Female	255 (44.5)	**0.010** ^b^
Male	318 (55.5)	
Sickle cell genotypes, n (%)		
HbAA	367 (82.7)	0.492^c^
HbAS	72 (16.2)	
HbSS	5 (1.1)	
Number of events in children, n (%)	15,006 (100)	
Temperature, °C	36.5 (0.6)	N/A
Afebrile, <37.5	13,704 (91.3)	**≤2.200E-16** ^a^
Febrile, ≥37.5	1,302 (8.7)	
Hemoglobin, (g/dL)	10.58 (1.99)	N/A
Parasite density, MPS/µL	7,980 (33,881)	N/A
Low (≤999), n (%)	399 (17.5)	**1.972E-260** ^a^
Medium (1,000–49,999), n (%)	1,496 (65.7)	
High (≥50,000), n (%)	382 (16.8)	
Geomean parasite density	7,303.6	N/A
Total *Plasmodium* cases, n (%)	2,277 (15.2)	
*Plasmodium falciparum troph*	2,261 (99.3)	**≤2.200E-16** ^a^
*Plasmodium malariae troph*	13 (0.6)	
Mixed infection (*Pf/Pm*)	3 (0.1)	
mRDT measurements, n (%)	9,958 (66.4)	N/A

Demographic and clinical characteristics of mother-child pairs (n = 573). Data are presented as median (interquartile range; IQR) unless stated otherwise. Statistical significance was determined using the

^a^ Chi-square goodness of fit.

^b^ Proportions test (binomial test for equal frequencies), and

^c^ Hardy Weinberg equilibrium. Bold indicates a statistically significant *p*-value after multiple test corrections using the Bonferroni-Holm method (familywise error rate, significance level at 0.050). MPS: malaria parasites, mRDT: malaria rapid diagnostic test, *troph* = trophozoites, *Pf/Pm; Plasmodium falciparum/Plasmodium malariae*.

### Demographic and clinical characteristics for concomitant microscopy (MPS) and mRDT events

The primary objective of this study was to assess the performance characteristics and antigenemia persistence associated with *Pf*HRP2-mRDT, using microscopy as the reference standard. To achieve this objective, only events that included both microscopy and mRDT were utilized (n = 9,958). Based on concomitant MPS/mRDT measures, events were stratified into four groups: MPS (+)/mRDT (+) [15.8%, n = 1,572], MPS (−)/mRDT (+) [29.2%, n = 2,907], MPS (+)/mRDT (−) [0.6%, n = 61], and MPS (−)/mRDT (−) [54.4%, n = 5,418]. The demographic and clinical characteristics for each category are shown in Table 2. The distribution of females and males was comparable across the groups (*p* = 0.176). Temporal temperatures at visits varied across the groups (*p* = 3.280E-86) with more febrile events (≥37.50°C) in the MPS (+)/mRDT (+) groups (*p* = 7.537E-129). Consistent with microscopy accurately detecting acute malaria infections, Hb levels differed across the groups (*p* = 7.525E-155) and were lower in the MPS (+)/mRDT (+) and MPS (+)/mRDT (−) categories. Comparison of the two MPS (+) groups revealed higher parasite and geometric mean densities in MPS (+)/mRDT (+) than MPS (+)/mRDT (−) group (*p* = 9.513E-09, and *p* = 0.018, respectively) with a higher percentage of low-density parasitemia (≤999 µL) in the MPS (+)/mRDT (−) group (*p* = 6.097E-08). Among the total MPS (+) events (n = 1,633), there were 1,620 infections due to *P. falciparum* and 13 attributable to *P. malariae*. The distribution of sickle cell genotypes differed across the groups (*p* = 2.048E-22), with a higher percentage of HbAS in MPS (−)/mRDT (+) and MPS (−)/mRDT (−), consistent with the protective nature in HbAS carriers [46, 47].

**TABLE 2:** Demographic and Clinical Characteristics for Concomitant Microscopy (MPS) and mRDT Events.

Concomitant Microscopy (MPS) and mRDT Events						
Characteristic	Total	MPS (+)/mRDT (+)	MPS (−)/mRDT (+)	MPS (+)/mRDT (−)	MPS (−)/mRDT (−)	*p*-value
No of events, n (%)	9,958 (100)	1,572 (15.8)	2,907 (29.2)	61 (0.6)	5,418 (54.4)	
Sex, n (%) Female Male	4,439 (44.58) 5,519 (55.12)	686 (43.6) 886 (56.4)	1,260 (43.3) 1,647 (56.7)	31 (50.8) 30 (49.2)	2,462 (45.4) 2,956 (54.6)	0.176^a^
Temperature, (^0^C)	36.5 (0.5)	36.6 (1.20)	36.5 (0.4)	36.8 (1.4)	36.5 (0.5)	**3.280E-86** ^b^
Afebrile, <37.5°C	9,071 (91.09)	1,125 (71.6)	2,759 (94.9)	37 (60.7)	5,150 (95.1)	**7.537E-129** ^a^
Febrile ≥37.5°C	887 (8.91)	447 (28.4)	148 (5.1)	24 (39.3)	268 (4.9)	
Hematological Parameter						
Hemoglobin, (g/dL)	10.6 (2.0)	9.9 (2.1)	10.2 (1.9)	9.7 (2.5)	10.9 (1.8)	**7.525E-155** ^b^
Parasitological Indices						
Parasite density, (MPS/µL)	6,607.8 (30,476.3)	7,030 (30,826.7)	-	957.0 (3,397.6)	-	**9.513E-09** ^c^
Low (≤999/µL), n (%)	319 (19.5)	288 (15.6)	-	31 (50.8)	-	**6.079E-08** ^a^
Medium (1,000–49,999/µL), n (%)	1,063 (65.1)	1,038 (66.1)	-	25 (41.0)	-	
High (≥50,000/µL), n (%)	251 (15.4)	246 (18.3)	-	5 (8.2)	-	
Geometric mean parasite density	3,526.7	21,886.2	-	11,708.7	-	**0.018** ^d^
*Plasmodium falciparum troph*	1,620 (16.3)	1,572 (100.0)	-	48 (78.7)	-	N/A
*Plasmodium malariae troph*	13 (0.1)	0.0	-	13 (21.3)	-	N/A
Genetic Variants						
Sickle cell genotype, n (%) HbAA	9,621 (100) 7,906 (82.2)	1,333 (87.0)	2,435 (86.0)	54 (90.0)	4,082 (78.6)	**2.048E-22** ^e^
HbAS	1,607 (16.7)	183 (12.0)	381 (13.5)	6 (10.0)	1,035 (19.9)	
HbSS	108 (1.1)	16 (1.0)	16 (0.5)	0 (0.0)	76 (1.5)	

Data are presented as counts, median (interquartile range; IQR), or mean (standard error of the mean; SEM). Statistical significance was determined using

^a^ Chi-square homogeneity test.

^b^ Kuskal-Wallis rank test.

^c^ Mann-Whitney U test (pairwise comparison).

^d^ Two-sample *t*-test, with equal variance, and

^e^ Fisher’s exact test. Bold indicates a statistically significant *p*-value at 0.05 after multiple test corrections using the Bonferroni-Holm method (familywise error rate). MPS: malaria parasites, mRDT: malaria rapid diagnostic test, *troph*: trophozoites.

### High sensitivities and moderate specificities of the mRDT

**FIGURE 5:**
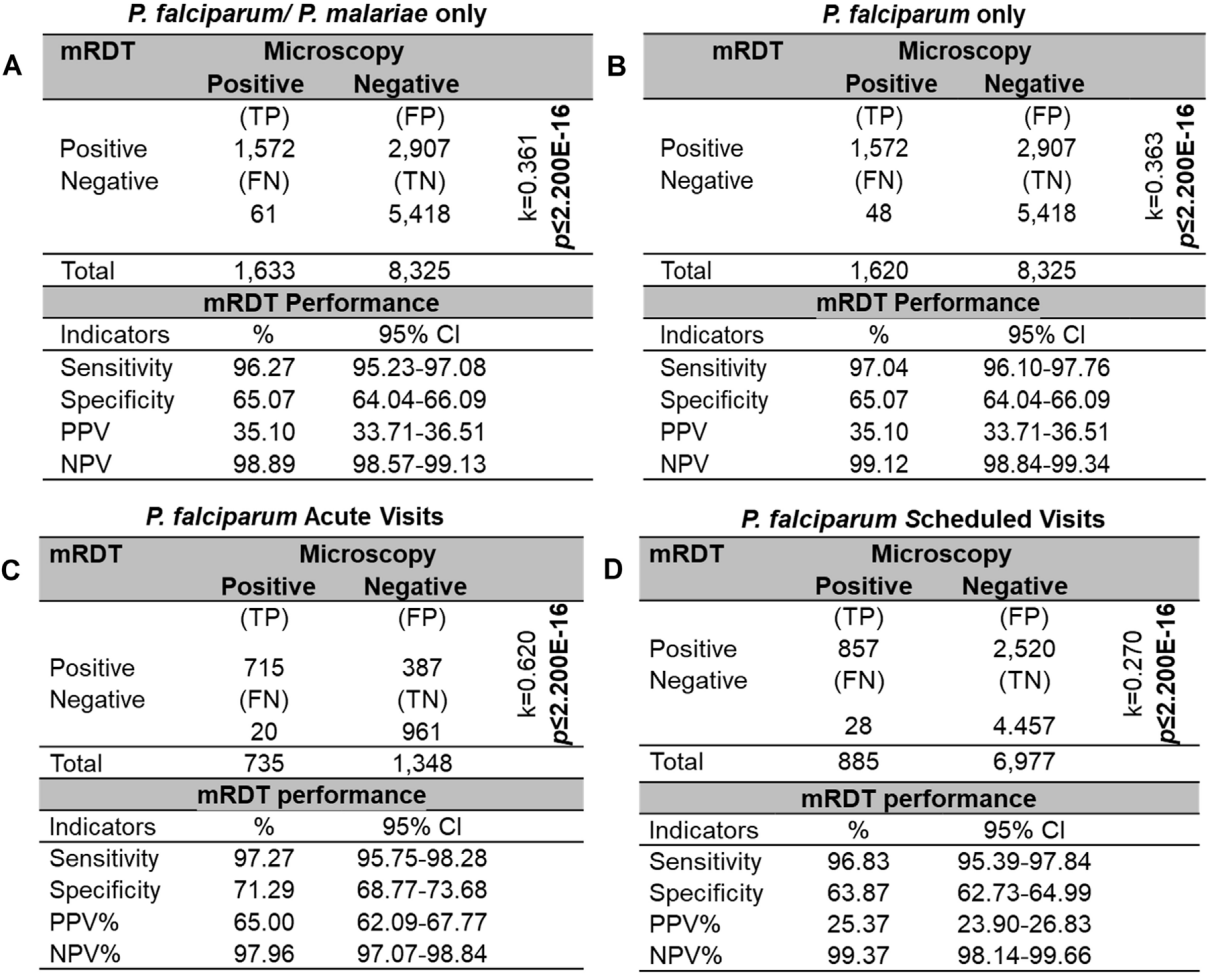
Sensitivity, specificity, and predictive values for mRDT measurements. Sensitivity, specificity, and predictive values for concomitant mRDT events (n = 9,958), using microscopy test (MPS) as the reference standard. Diagnostic measures are reported for four different scenarios: **(A)** *P. falciparum* (n = 1,620) and/or *P. malariae* (n = 13). **(B)** *P. falciparum* only. **(C)** *P. falciparum* only at acute visits. **(D)** *P. falciparum* only at scheduled follow-up visits. Statistical significance was determined using a Chi-square homogeneity test with a significance level of *p* ≤ 0.050. Inter-rater agreement was calculated using Cohen’s Kappa (k). TP: True positives, FP: False positives, FN: False negatives, TN: True negatives, PPV: Positive predictive value, NPV: Negative predictive value.

The sensitivities, specificities, and predictive values of mRDTs can vary based on geographical regions, parasite genetic diversity, and levels of parasitemia [47]. Therefore, the diagnostic characteristics were evaluated in a high transmission region in western Kenya. Using microscopy as the reference standard, there were 1,572 true positives (TP), 2,907 false positives (FP), 61 false negatives (FN), and 5,418 true negatives (TN) for the combined *P. falciparum* and *P. malariae* infections (*p* < 2.20E-16, k = 0.361, Figure 5A). The mRDT demonstrated a high sensitivity of 96.27% (95%CI: 95.23–97.08) and a moderate specificity of 65.07% (95%CI: 64.04–66.09). The PPV was low at 35.10% (95%CI: 33.71–36.51), whereas the NPV was high at 98.89% (95%CI: 98.57–99.13). When considering only *P. falciparum* cases (Figure 5B), sensitivity showed a slight increase to 97.04% (95%CI: 96.10–97.76) and NPV to 99.12% (95%CI: 98.84–99.34), while specificity and PPV remained unchanged.

Performance characteristics were further evaluated by stratifying events based on visit type (i.e., acute *vs.* scheduled follow-up), excluding *P. malariae* cases. During acute visits, the mRDT demonstrated a sensitivity of 97.27% (95%CI: 95.75–98.28) and specificity of 71.29% (95%CI: 68.77–73.68), with a PPV of 65.00% (95%CI: 62.09–67.77) (Figure 5C). In contrast, for scheduled follow-up visits, while sensitivity remained high at 96.83% (95%CI: 95.39–97.84), specificity decreased to 63.87% (95%CI: 62.73–64.99), resulting in a lower PPV of 25.37% (95%CI: 23.90–26.83) (Figure 5D). These results are supported by the 2.3-fold higher Cohen’s kappa value (k) observed during acute visits, indicating a more substantial agreement between the mRDT and microscopy than in scheduled follow-ups. Importantly, the NPV remained consistently high, exceeding 97% for both visit types, demonstrating the mRDTs reliability in ruling out malaria infections regardless of visit type.

### Longitudinal measures indicate prolonged HRP2 antigenemia

**FIGURE 6:**
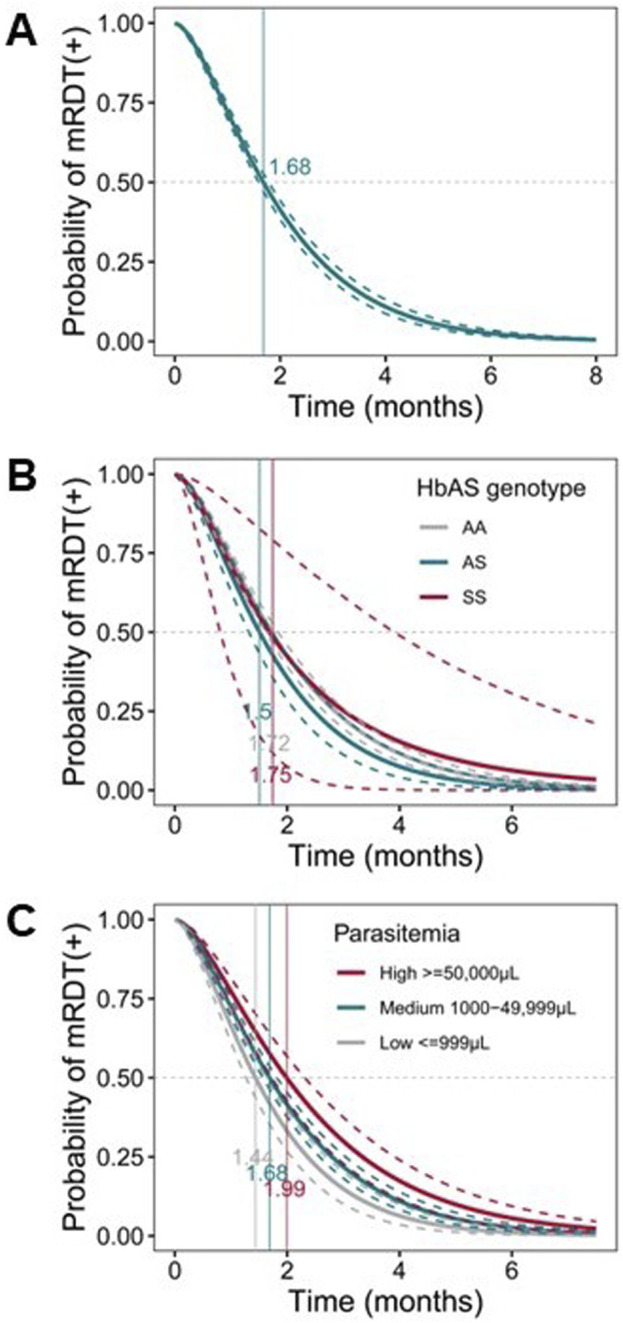
Probability of mRDT positive post-infection. Parametric, interval-censored Cox proportional hazards models with a gamma-exponential distribution were used to evaluate *Pf*HRP2 antigenemia persistence. The dataset used for this analysis was pre-processed to include only cases where both microscopy and mRDT were consistently positive at least once (n = 291 children; 1,294 events). The x-axis represents the time in months, and the y-axis shows the probability of mRDT remaining positive after parasite clearance. The curved dotted lines represent upper and lower confidence bounds, and the solid lines depict the decay probabilities of each group. Dotted gray lines at y = 0.50 represent the 50% probability. Vertical lines depict the median time to mRDT negative. **(A)** Survival curve representing the probability of mRDT (+) post-infection. **(B)** Survival curve representing the probability of mRDT (+) post-infection with parasitemia levels as covariates [≤999/μL (grey); medium [mid, 1,000–49,999/μL (turquoise)]; and high [≥50,000/μL (maroon)]. **(C)** Survival curve representing the probability of mRDT (+) post-infection with HbS genotypes as covariates: AA (grey), AS (turquoise), and SS (maroon).

The rate of *Pf*HRP2 antigen decay following parasite clearance significantly impacts mRDT performance, influencing the sensitivity, specificity, and overall efficiency of the test [47]. A Cox proportional hazards model for ordered events was utilized to evaluate the decay kinetics in the longitudinal cohort. To ensure accurate analysis of malaria episodes [MPS (+)] and *Pf*HRP2 decay, the dataset was filtered to include only cases where microscopy and mRDT were consistently positive at least once. Visits before the first confirmed malaria episode and those with inconsistent diagnostic results were excluded. Intermittent positive results focused only on visits relevant to malaria progression and resolution. This approach ensured the analysis reflected the actual dynamics of malaria infection and *Pf*HRP2 persistence in the pediatric cohort. After rigorous filtering steps, the data included 291 children with 1,294 events. The positivity rate of *Pf*HRP2 antigenemia remained high (80–100%) within the first month and declined significantly over time (*p* ≤ 2.200E-16). The time for the positivity rate of *Pf*HRP2 antigenemia to reach 0.5 (i.e., 50% of *Pf*HRP2 decay) was 1.68 months (51.14 days), respectively (Figure 6A), indicating the persistence of *Pf*HRP2 in the bloodstream of the pediatric cohort. Stratification of children with initial parasite levels revealed that those with low parasite densities had a significantly faster *Pf*HRP2 antigen decay compared to those with either high initial parasite densities (*p* = 0.001) or medium parasite densities (*p* = 0.001). The 50% of *Pf*HRP2 decay for the three groups were 1.44 mos. (43.83 days), 1.99 mos. (60.58 days) and 1.68 mos. (51.14 days) respectively (Figure 6B).

Additional analyses revealed that sickle cell genotypes partially influence *Pf*HRP2 antigen decay rates (Figure 6C). Specifically, 50% of *Pf*HRP2 decay takes 1.72 mos. (51.75 days), 1.51 mos. (45.66 days) and 1.75 mos. (53.27 days) for malaria patients with HbAA, HbAS, and HbSS genotypes, respectively. Relative to the HbAA reference group, carriers of the HbAS genotype had a non-significantly shorter decay rate (*p* = 0.088), but no difference was observed for carriers of the HbSS genotype (*p* = 0.991). These analyses reveal that antigenemia persists for a prolonged period and is influenced by the initial parasite density and potentially, sickle cell genotypes.

### Validation of mRDT performance in a short-term (14-day) febrile cohort

**FIGURE 7:**
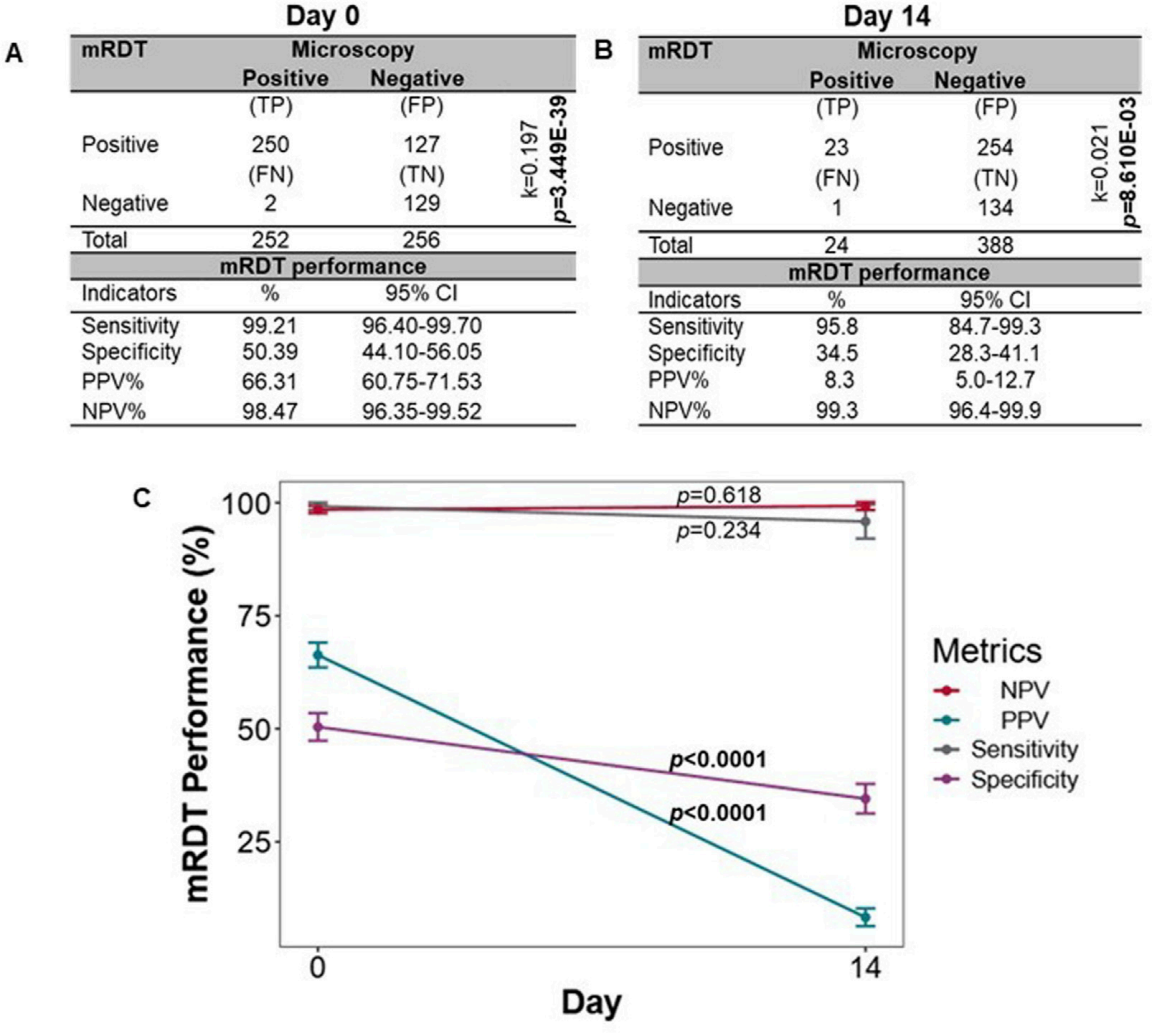
Sensitivity, specificity, and predictive values for mRDT measurements on day 0 and day 14. The mRDT performance was evaluated using microscopy as the reference standard. **(A)** Sensitivity, specificity, and predictive values for mRDT measurements on day 0 (n = 508 children)*.* **(B)** Sensitivity, specificity, and predictive values for mRDT measurements on day 14 (412 events). For A and B, statistical significance was determined using Fisher’s exact test, and the level of agreement between the two diagnostic tests was computed using Cohen’s Kappa statistics (k). **(C)** Comparison metric of the sensitivities, specificities, and predictive values between day 0 and day 14. Statistical significance was determined using Fisher’s exact test. TP: True positives, FP: False positives, FN: False negatives, TN: True negatives, PPV: Positive predictive values, NPV: Negative predictive value.

To further explore the performance of the mRDT, we utilized an independent dataset from a prospective febrile cohort with concomitant microscopy and mRDT results on day 0 (acute illness, n = 508) and day 14 (well-visit, n = 412). The demographic and clinical characteristics of the children at enrollment (day 0) are presented in Table 3. As expected during acute or recent malaria infections, Hb concentrations differed across the four groups (*p* = 0.025) and were highest in the MPS (−)/mRDT (−) group. The distribution of sickle cell genotypes also significantly differed (*p* = 1.000E-05) with the highest percentage of HbAS carriers in the MPS (−)/mRDT (−) group. Comparison of mRDT performance at days 0 and 14 revealed that acute disease (day 0) was characterized by higher sensitivity [99.21% (95%CI: 96.40–99.70) vs. 95.83% (95%CI: 84.68–99.27)], specificity [50.47% (95%CI: 44.10–56.05) vs. 34.54% (95%CI: 28.28–41.01)], and PPV [66.34% (95%CI: 60.75–71.53) vs. 8.30% (95%CI: 4.98–12.73%) (Figures 7A,B). The NPV remained high on both visit days (>98%). These results indicate that the mRDT is more effective at detecting and ruling out a malaria infection during acute disease (day 0) and follow-up visits, respectively.

**TABLE 3:** Demographic and Clinical Characteristics for Concomitant Microscopy (MPS) and mRDT Categories in the Febrile Cohort.

Concomitant Microscopy (MPS) and mRDT Measures						
Characteristic	Total	MPS (+)/mRDT (+)	MPS (−)/mRDT (+)	MPS (+)/mRDT (−)	MPS (−)/mRDT (−)	*p*-value
Sample size, n (%)	508 (100)	250 (49.2)	127 (25.0)	2 (0.4)	129 (25.4)	
Sex, n (%) Female Male	237 (46.7) 271 (53.3)	116 (46.4) 134 (53.6)	61 (48.0) 66 (52.0)	2 (100.0) 0.0	58 (45.0) 71 (55.0)	0.581^a^
Age at enrollment (months)	21.7 (26.6)	20.8 (22.73)	25.2 (30.1)	10.5 (N/A)	16.1 (26.7)	0.004^b^
Temperature, (^0^C)	38 (0.7)	38.0 (0.6)	38.0 (0.6)	39.2 (N/A)	38.0 (0.8)	0.137^b^
Afebrile, <37.5°C	37 (7.3)	16 (6.4)	10 (7.9)	0.0	11 (95.1)	0.756^a^
Febrile, ≥37.5°C	471 (92.7)	234 (93.6)	117 (92.1)	2 (100.0)	118 (4.9)	
Hematological Parameter						
Hemoglobin, (g/dL)	7.6 (4.4)	7.3 (3.8)	7.0 (4.5)	7.1 (N/A)	8.9 (5.2)	**0.025** ^b^
Parasitological Indices						
Parasite density, (MPS/µL)	5,514 (39,714)	5,514 (39,506)	-	77,886 (N/A)	-	**3.256E-94** ^c^
Low (≤999/µL), n (%)	65 (25.8)	65 (25.8)	-	0.0	-	N/A
Medium (1,000–49,999/µL), n (%)	132 (52.4)	131 (52.4)	-	1 (50.0)	-	N/A N/A
High (≥50,000/µL), n (%)	55 (21.8)	54 (21.8)	-	1 (50.0)	-	
Geometric mean parasite density	0.0	6,147	-	14,580	-	N/A
*Plasmodium falciparum troph*	250 (99.6)	250 (100)	-	0 (0.0)	-	N/A
*Plasmodium malariae troph* Mixed Infection (*Pm/Pf*)	1 (0.2) 1 (0.2)	0 (0.0) 0.0	- -	1 (50.0) 1 (50.0)	- -	N/A N/A
Genetic Variants						
Sickle cell genotypes, n (%) HbAA	492 (100) 377 (76.6)	208 (84.9)	96 (79.3)	2 (100.0)	71 (57.2)	**1.000E-05** ^a^
HbAS	56 (11.4)	22 (9.0)	11 (9.1)	0.0	23 (18.5)	
HbSS	59 (12.0)	15 (6.1)	14 (11.6)	0.0	30 (24.3)	

Data are presented as median (interquartile range; IQR), unless otherwise indicated. Statistical significance was determined using

^a^ Fisher’s exact test.

^b^ Kruskal-Walli’s rank test, or

^c^ Mann-Whitney U test (pairwise comparison). The boldface indicates a statistically significant *p*-value at a 0.05 threshold after multiple test corrections using the Bonferroni-Holm method (familywise error rate). MPS: malaria parasites, mRDT: malaria rapid diagnostic test, *troph*: trophozoites, *Pm/Pf*: *Plasmodium falciparum/Plasmodium malariae*.

Next, a direct comparison was made of the mRDT performance between days 0 and 14 (Figure 7C). Although higher on day 0, sensitivity did not differ between days 0 and 14 (*p* = 0.234), whereas specificity was significantly higher on day 0 than day 14 (*p *< 0.0001). The PPV was higher on day 0 compared to day 14 (*p *< 0.0001), highlighting the mRDT’s reliability in detecting true malaria cases at initial diagnosis. However, the NPV remained consistently high on both days (*p* = 0.618), exceeding 98%, demonstrating the test’s continued reliability in ruling out malaria infection when test results are negative. These results illustrate the utility of the mRDT’s diagnostic performance during acute malaria episodes and the potential for increased false positives during short-term follow-up assessments.

## Discussion

Rapid and reliable diagnostic methods are crucial for effective disease management in malaria-endemic regions, especially where *P. falciparum* transmission is high. Accurate and timely diagnosis is critical to guide appropriate treatment, especially in children under five, who are most vulnerable to severe malaria complications [3, 10]. In resource-limited settings where microscopy is sometimes not available, mRDTs have become essential tools, enabling timely diagnosis and treatment decisions [3, 7, 10, 11]. Our study provides a comprehensive evaluation of the diagnostic performance of *Pf*HRP2-based mRDTs and antigenemia persistence of *Pf*HRP2 in a *P. falciparum* holoendemic region of Kenya, contributing to a better understanding of their strengths and limitations in both acute and follow-up scenarios.

Findings from this study confirmed the high sensitivity (97.04%) and negative predictive value (NPV >99%) of the mRDTs in patients with *P. falciparum*, reinforcing their utility as frontline diagnostic tools in resource-constrained settings [48–50]. Our results are consistent with previous studies demonstrating their reliability in detecting *P. falciparum* infections, even in cases of low parasitemia [6, 15, 35]. However, the moderate specificity (65.07%), particularly during longitudinal follow-up visits, represents significant limitations, illustrating that mRDTs are not useful diagnostic tools for following the patients’ malaria status after initial diagnosis. Reduced specificity is likely attributed to the persistence of *Pf*HRP2 antigen in the bloodstream after parasite clearance [37, 47, 51–53].

The performance of mRDTs can vary depending on parasite species, the target gene(s) deletion, and the timing of persistent antigenemia [47, 54]. Our study expands current knowledge in real-world settings by demonstrating how the type of patient visits influences mRDT performance. Performance varied significantly by visit type, with better specificity and positive predictive value (PPV 65.00%) during acute visits when patients presented with higher parasitemia and clinical symptoms in the longitudinal study and validation cohort. These findings align with evidence showing that higher parasite densities improve the accuracy of mRDTs [18, 55, 56]. Conversely, during scheduled follow-up visits after administration of antimalarial therapy, specificity and PPV declined substantially, likely because *Pf*HRP2 antigens persisted from prior infections. This highlights the challenge of using mRDTs for post-treatment monitoring, particularly in high-transmission areas with persistent exposure, since positive results may not necessarily indicate active infection but rather residual antigenemia.

Notably, the mRDT specificities in the longitudinal mother-child and validation (acute febrile) cohorts were lower than the sensitivities, potentially due to recent antimalarial treatment that could reduce detectable parasites by microscopy but have little influence on the persistence of circulating antigens measured by the mRDT. While microscopy remains the gold standard for malaria diagnosis, the mRDT’s utility lies in its rapid and accessible nature, particularly in resource-limited settings [12]. This moderate agreement between mRDTs and microscopy highlighted the need for confirmatory testing to improve malaria control and patient outcomes in endemic regions.

The sensitivity and specificity were validated by comparing results from the mother-child longitudinal birth cohort to those of an independent acute febrile cohort enrolled on day 0 (n = 508) and followed up on day 14 (n = 412) in the identical community. The mRDT demonstrated better sensitivity and specificity on day 0 than on day 14 (well-visit), confirming the trends observed in the mother-child cohort during acute and follow-up visits. For clinical relevance, the diagnostic performance of mRDTs should be interpreted in the context for which they are most aptly applied: evaluating febrile illness in symptomatic individuals. To address this, we conducted targeted performance analyses of mRDTs at acute pre-treatment visits in symptomatic children in both the longitudinal and the short-term febrile cohorts. These analyses reflect real-world clinical application. Sensitivity was high in both settings, 97.27% in the longitudinal cohort and 99.21% in the short-term cohort, while specificity was lower, at 71.29% and 50.39%, respectively. Factors that may account for lower specificity include submicroscopic infections undetected by microscopy that yield a positive mRDT (apparent false positives). Moreover, residual *Pf*HRP2 antigenemia from recent infections may persist in holoendemic settings despite parasite clearance, yielding discordant results. The acute febrile cohort’s even lower specificity may be due to the likelihood of this cohort having a broader range of severe, non-malarial conditions and more recent treatment since they came directly from the community and were not part of a longitudinal study. The Cohen’s kappa value observed during acute visits was 2.3 times higher than at scheduled follow-ups, indicating substantially greater agreement between mRDT and microscopy when used in symptomatic, pre-treatment cases. The consistency of findings across both studies strengthens the generalizability of our observations.

The longitudinal study design of the mother-child cohort with 36 mos. of follow-up allowed for a thorough evaluation of *Pf*HRP2 antigen decay kinetics. We found the time for positivity rate of *Pf*HRP2 antigenemia to reach 0.5 after parasite clearance was 1.68 months (i.e., 51.14 days). A comprehensive analysis using a Bayesian survival model, which aggregated data from multiple published studies, predicted the clearance time of the *Pf*HRP2 antigen following malaria treatment. The model estimated that 95% of *Pf*HRP2 tests are negative within 36 days (95%CI: 21–61 days) after treatment [23]. Our findings fall within the upper limit of this range, suggesting a similar, albeit slightly prolonged, antigen clearance timeline in our cohort. This could be explained by the rigorous study design and filters employed during this analysis, which enabled detailed tracking of antigen decay kinetics in the current study.

Stratification of the cohort by initial *P. falciparum* parasite density revealed that higher parasite densities were associated with prolonged antigen persistence, consistent with findings from previous studies in Uganda [60]. This prolonged persistence underscores the challenge of using mRDTs in high-transmission settings, where high parasite burdens are more frequent [19]. In addition, we explored the impact of sickle cell status on antigen decay. Although not statistically significant, results suggested trends of slower *Pf*HRP2 antigen decay rates in individuals with HbAS and HbSS compared to those with the HbAA genotype. These findings suggest a potential influence of host genetics on antigen clearance and require further investigation, particularly in regions where the sickle cell trait is prevalent.

The issue of *Pf*HRP2 gene deletions presents an additional diagnostic challenge that can lead to false-negative mRDT results. Such gene deletions have been increasingly reported in malaria-endemic regions; however, the prevalence is relatively low in Kenya compared to other countries such as Eritrea and Djibouti [17, 54, 58–61]. However, we are currently investigating deletions of the target gene in the false negatives detected in this study. The false negative results in this study are likely due to low parasite densities (957 MPS/μL) in the MPS (+)/MPS (−) group in which the mRDT failed to detect the antigen. Additional reasons for false-negative results in the MPS (+)/mRDT (−) group may be due to the timing of infection, in which very early infections were detected by microscopy, but before *Pf*HRP2 is secreted at detectable levels. It is unlikely that false negatives were due to a prozone effect (highly excessive antigen) since children with very high parasite densities fell withing the MPS (+)/mRDT (+) group.

In conclusion, our findings emphasize the utility of *Pf*HRP2*-*based mRDTs as a valuable tool for diagnosing acute malaria in children under five, where sensitivity and NPV were high. However, reduced specificity during follow-up visits, due to *Pf*HRP2 antigen persistence, poses significant challenges for ongoing patient monitoring. This can result in overtreatment and misdiagnosis, underscoring the importance of integrating confirmatory diagnostics, such as microscopy or molecular tests, to improve diagnostic accuracy and patient outcomes. The strengths of this study include the extensive follow-up of children, which allows for an unbiased assessment of *Pf*HRP2 antigen decay rates and the validation of the results using an independent cohort. A potential limitation of the study is the use of microscopy as the reference standard for evaluating mRDT performance, since submicroscopic infections would go undetected. Future studies incorporating molecular diagnostics could provide more precise estimates of mRDT performance. Another potential limitation is that we did not directly measure *Pf*HRP2 protein levels using ELISA or Western Blot due to limited sample volume. However, our transcriptomic profiling of *P. falciparum* in a subset of patients provided indirect evidence of the antigenemia detected by the mRDT. *Pf*HRP2 was among the most highly expressed *P. falciparum* genes in Day 0 (pre-treatment) samples and was absent at Day 14 (well visit) in aparasitemic children. This suggests that persistent mRDT positivity reflects residual circulating antigen rather than active infection. Collectively, *Pf*HRP2-based mRDTs are valuable diagnostic tools at initial presentation, where sensitivity and specificity are optimal, but antigen persistence limits their effectiveness in follow-up assessments. Understanding these limitations in the context of confirmatory testing is crucial for optimal patient management and malaria control efforts to reduce the disease burden in high-transmission regions.

## Data Availability

The raw data supporting the conclusions of this article will be made available by the authors, without undue reservation.
